# Ultrasound Assessment of the Abdominal Wall Muscles in Women with and without Primary Dysmenorrhea: A Cross-Sectional Study

**DOI:** 10.3390/diagnostics10030166

**Published:** 2020-03-18

**Authors:** Carlos Romero-Morales, Mónica de la Cueva-Reguera, Beatriz Miñambres-Vallejo, Beatriz Ruiz-Ruiz, César Calvo-Lobo, Israel Casado-Hernández, Daniel López-López, Vanesa Abuín-Porras

**Affiliations:** 1 Faculty of Sport Sciences, Universidad Europea de Madrid, Villaviciosa de Odón, 28670 Madrid, Spain; carlos.romero@universidadeuropea.es (C.R.-M.); monica.delacueva@universidadeuropea.es (M.d.l.C.-R.); Beatriz.minambres@gmail.com (B.M.-V.); beatriz.ruiz@universidadeuropea.es (B.R.-R.); vanesa.abuin@universidadeuropea.es (V.A.-P.); 2 Facultad de Enfermería Fisioterapia y Podología, Universidad Complutense de Madrid, 28040 Madrid Madrid, Spain; cescalvo@ucm.es (C.C.-L.); israelcasado@yahoo.es (I.C.-H.); 3 Research, Health and Podiatry Group, Department of Health Sciences, Faculty of Nursing and Podiatry, Universidade da Coruña, 15403 Ferrol, Spain

**Keywords:** primary dysmenorrhea, ultrasound imaging, abdominal wall

## Abstract

Background: Primary dysmenorrhea (PD) is one of the most common gynecological disorders in women of reproductive age that may affect quality of life. It is believed that the underlying cause of PD may be the excessive production of prostaglandins (PGs), however, between 20%–25% of women with PD did not respond to pharmacological treatments, showing that nociceptive mechanisms underlying PD are still not understood. The purpose of this study was to measure and compare, through the use of ultrasound imaging, the thickness at rest of the abdominal wall, as well as the interrecti distance (IRD), in women with and without PD. Methods: A cross-sectional study has been performed using ultrasound imaging (USI) to measure the resting thickness of the external oblique (EO), internal oblique (IO), transversus abdominis (TrAb), rectus abdominis (RA), as well as the IRD in a sample of 39 women, 19 with PD and 20 without PD (median ± IR age: 20 ± 4 and 22.5 ± 7 years, respectively). Results: Findings of muscular thickness did not reveal statically significant differences (*p* < 0.05) in EO, IO, TrAb, RA, and the IRD between the PD group and control group. Conclusions: These findings suggest that the thickness of the abdominal wall is not associated with PD.

## 1. Introduction

Pelvic pain is a debilitating condition commonly experienced by women with a relevant impact at the emotional, functional and economic level [1]. Dysmenorrhea is one of the underlying mechanisms of gynecological origin that leads to this chronic pelvic pain [2]. In primary dysmenorrhea (PD), recurrent menstrual pain occurs in the absence of identifiable pelvic pathology. Worldwide, it is estimated that between 20%–90% of adolescents and more than 50% of adult women suffer from PD [3,4], and among these women, 10% to 20% report their symptoms as moderate or severe. In addition, Polat et al. argued that PD could interfere with usual activities from one to three days, being considered as the main cause of absenteeism from school and work in the short term [4]. Nevertheless, the specific cause of PD has not yet been found and there are only theories and hypotheses that seek to find a causal explanation. The most widely accepted explanation attributes the cause of PD to the pathophysiological changes during the post-ovulatory period, and during this period, there is an overproduction of uterine prostaglandins which leads to myometrial hypercontractility [5,6]. In addition, if changes in neuroendocrine and hormonal secretions persist, secondary pathologies could occur, such as endometriosis [7,8,9].

Several authors argued that PD may contribute to the development of a chronic pelvic pain disorder [1,2], showing high comorbidity with other chronic pain conditions, such as irritable bowel syndrome, migraines and fibromyalgia [10]. Nevertheless, since only 75%–80% of women treated with pharmacology experienced relief from PD symptoms, it is suggested that the nociceptive mechanisms underlying PD are not yet understood [11].

Kim et al. [12] reported that women who showed an imbalance in the lumbopelvic system experienced greater menstrual pain. A possible explanation for this could be that the change in the position of the uterus, due to the imbalance of the pelvis, caused an excessive amount of PGs to be secreted [13]. In addition, Blakey et al. [14] assume that PD can be caused by a dyssynergy between the muscles of the pelvic cavity and the soft tissues. Some treatments proposed to manage PD, such as heat, massage, Transcutaneous electrical nerve stimulation (TENS) or vertebral manipulations, could plausibly address the dysfunction of the abdominal muscles [11,15,16]. Nevertheless, few research studies have been conducted establishing associations between the abdominal muscles and PD.

The use of ultrasound imaging (USI) has been described as a useful method to assess and quantify the musculoskeletal and soft tissue complex. USI can be considered an effective, safe, cost-effective and easy accessible imaging technique that makes it possible to rapidly evaluate the morphology and pathomorphological changes of various organs and soft tissues [17,18,19]. Prior studies about USI aimed to detect atrophy in lumbar muscles in subjects with lower back pain. In this line, USI applications with respect to many other muscles in the abdomen and extremities continue to be investigated in different populations [20,21]. In addition, due to the depth of the abdominopelvic muscles, few tools may measure in a reliable and non-invasive way the morphological characteristics such as length, depth, diameter, cross-sectional area, volume and rotation angles, as well as the changes in these characteristics and their impact on the associated structures, such as fascia and organs such as the bladder or the uterus, as USI can [18,22].

Based on prior studies about the relationship between the thickness of the abdominal wall muscles and PD, the present cross-sectional observational study aimed to assess and quantify the thickness of the abdominal wall musculature—external oblique (EO), internal oblique (IO), transverse abdominis (TrAb), rectus abdominis (RA)—and the inter-rectus distance (IRD) between women with and without PD. We hypothesized that thickness differences could be shown for the abdominal wall muscles between the two groups, which could be reduced in the group with PD.

## 2. Materials and Methods

### 2.1. Study Design

A prospective, cross-sectional, observational study was performed at the European University of Madrid Research Lab from October 2018 to June 2019, following the recommendations of the STROBE Declaration (Strengthening the Reporting of Observational Studies in Epidemiology) [23].

### 2.2. Ethical Considerations

This study was designed in accordance with the fundamental principles for clinical research in humans described in the Declaration of Helsinki [24], and it was examined and approved by the Ethics Committee of the European University of Madrid, Spain [(29/February/2019) CIPI/19/003]. Before inclusion, all participants were provided with a sufficient description of the purpose and procedure of the study, obtaining their signature through the informed consent form.

### 2.3. Participants

A total sample of 39 women from 18 to 45 was recruited for the present study and divided into two groups: DP group (*n* = 19) and a control group (*n* = 20). For the DP group participants were added if they were women of childbearing age (18–45 years) [13,15], nulliparous, diagnosed with PD according to the Dysmenorrhea Consensus Guideline [15], with regular menstrual cycles (28 ± 7 days) [12,13,15], and with moderate or severe menstrual pain (more than 50 mm in the visual analog scale (VAS) [13,15]. The control group was composed of women with no history of menstrual pain who were not diagnosed with PD. The exclusion criteria for both groups were as follows: women diagnosed with secondary dysmenorrhea, regular use of any pharmacological treatment for PD (analgesics, nonsteroidal anti-inflammatory drugs (NSAIDs), etc.), administration of contraceptives orally or through any other vaginal or intradermall system, and previous gynecological and/or abdominal interventions [12,13,15].

G*Power software was employed for the sample size calculation by the difference between the DP group and control group using the TrAb thickness (mm) variable of a pilot study (*n* = 16) divided in two groups (mean ± SD): 8 subjects with PD (0.38 ± 0.10) and 8 subjects for the healthy group (0.30 ± 0.07). For the sample size calculation, a power of 0.80, an α error of 0.05 and an effect size of 0.92 with a 1-tailed hypothesis was employed. In conclusion, a sample of 32 was calculated. Nevertheless, we could include a sample of 39 individuals.

### 2.4. Outcome Measurements

**Figure 1:**
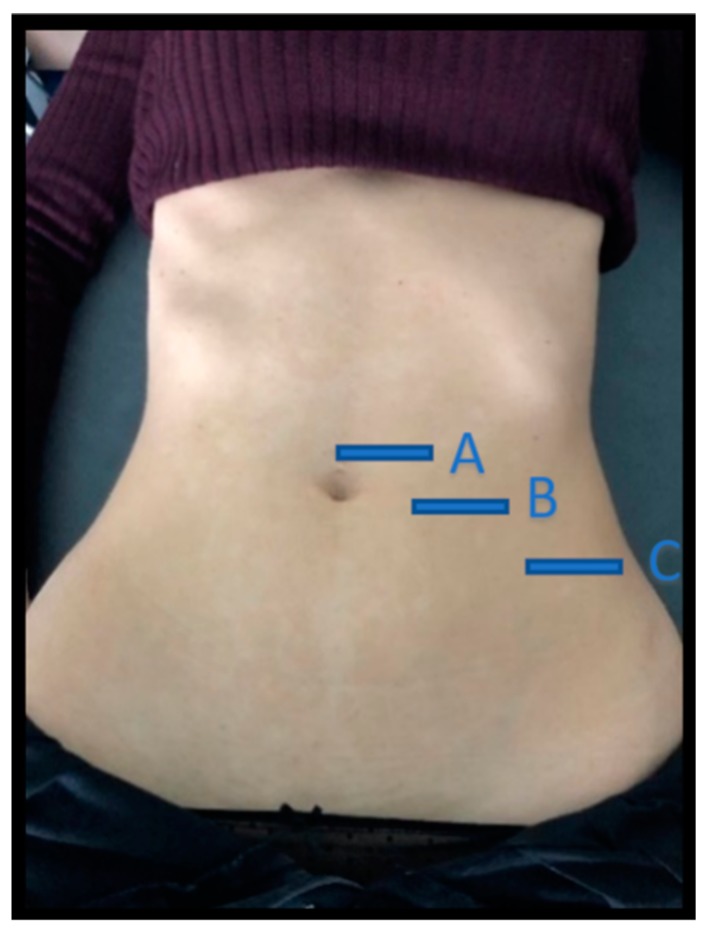
Location of the three abdominal imaging points, from lateral to medial: (A) midway between the iliac crest and the lower edge of the ribcage, in the axillary midline for the EO, IO, and TrAb; (B) over the midpoint of the AR muscle, at the level of the umbilicus; (C) in the midline of the abdomen just above the umbilicus for the IRD. EO—external oblique; IO—internal oblique; TrAb—transverse abdominal; AR—abdominis rectus; IRD—inter-rectus distance.

A high-quality ultrasound system (LOGIC^R^ S7 R3 XDclear, GE Healthcare, Milwaukee, WS, USA) was used in B mode, with a 5–15 MHz linear transducer (GE ML6–15; 44 mm field of view). The ultrasound images were obtained with the participants placed in supine position with the hips and knees bent and the arms alongside the body. The operator was positioned laterally to the right of the participant [18,25] (Figure 1).

**Figure 2:**
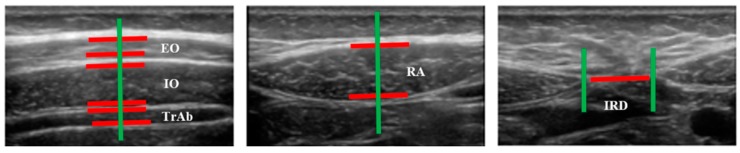
Measurements of muscle thickness and the IRD of the abdominal wall.

Images of three abdominal points were recorded according to Whittaker’s et al. [25] recommendations, obtained through palpation, which showed excellent intra and inter-rater reliability (ICC = 0.92–0.99). For the EO, IO and TrAb muscles, the transducer was placed between the iliac crest and the lower edge of the rib cage, in line with the axillary midline. For the RA muscle, the transducer was placed at the midpoint of the RA muscle at the umbilicus level [25]. Finally, for the IRD the transducer was placed just under the umbilicus [25]. Muscle thickness was defined as the distance amongst the edges of each muscle. IRD was designated as the distance between both RA, as argued by Whittaker et al. [18]. Image measurements were carried out using ImageJ software (version 2.0; US National Institutes of Health, Bethesda, MD, USA). The mean of three repeated measurements was collected. Each ultrasound image was captured, keeping the transducer in the same position and with the same pressure (only the weight of the transducer), at the end of exhalation, determined by visual inspection (Figure 2). All measures were carried out by a single specialized physical therapist with five years of USI experience.

### 2.5. Statistical Analysis

Statistical analysis was performed using the SPSS software for Windows (IBM SPSS Statistics for Windows; IBM Corp; Armonk, NY, USA). All data were analyzed with a 95% confidence interval (CI) (error α of 0.05) and a desired power of 80% (error β of 0.2). First, the normality assessment was carried out using the Kolmogorov-Smirnov test. Second, descriptive statistics were calculated for the total sample as a whole, as well as for both groups separately, including measures of central tendency and their dispersion ranges using the mean and standard deviation (SD) to describe parametric data, and the median and interquartile range (IR) to describe non-parametric data. Finally, a comparative analysis between the PD group and the healthy group was performed. The parametric data was analyzed using a Student’s t-test for independent samples, while for the non-parametric data, the U-test from Mann-Whitney was used. In addition, the Levene’s test was employed to evaluate the equality of variances.

## 3. Results

Regarding Table 1, no statistically significant differences (*p* > 0.05) were found between groups with PD and without PD based on the age (*p* = 0.647), height (*p* = 0.134), weight (*p* = 0.322), or BMI (*p* = 0.881) of the participants.

In addition, as shown in Table 2, there were not statistically significant group differences (*p* > 0.05) for ultrasound measurements of abdominal wall muscles (TrAb, IO, EO, RA) and there were no significant differences between groups. Considering the IRD, no statistically significant differences (*p* = 0.098) were found between women with PD (mean: 0.96 ± 0.48, 95% CI) and healthy women (mean: 1.26 ± 0.59, 95% CI).

## 4. Discussion

In this preliminary study, we sought to examine the relationship between the thickness of the abdominal musculature and the IRD in PD. The results did not reveal statistically significant differences between the groups in the thickness of TrAb, IO, EO and RA nor the IRD. However, the study conducted by Kim et al. [26] about the effects of lumbopelvic alignment and the thickness of the abdominal musculature in subjects with PD found differences between the PD group and the control group, reporting a decrease in the thickness of the TrAb and the IO for women with PD. In addition, the authors explained that disturbances in the muscular system resulting in instability may cause PD. Current literature research about the relationship between pain and musculoskeletal disorders in patients with pelvic floor disorders, such PD. For example, in the study performed by Kim et al. [26] the basal level of pain was 8 mm/10 in the VAS scale for both groups. Thus, one hypothesis could be that in patients with any level of pain, it would be more likely to find a thickness difference in abdominal muscles and that would be the reason of the discrepancy of the results, which would lead us to reflect on the role of nociceptive processing in skeletal imbalances and PD [27]. Therefore, pain intensity could be considered a determinant factor to evaluate in patients with PD. Further research is needed to improve quality of life, sexual function and pain intensity in women with pelvic floor disorders, for example an etonogestrel implant for the levonorgestrel-releasing intrauterine system seems to be an effective method in the treatment of women with dysmenorrhea [28,29].

In addition, according to the study by Lim et al. [30], kinesiotaping and spiral taping were effective in the treatment of PD in women with a VAS of five or higher. The effect of tape on PD could be due to the normalizing effect of imbalances in antigravity muscles, a hypothesis also supported by the study of Kim et al. [12].

However, Whittaker et al. [31], in their study on the USI evaluation of the abdominal wall taking into account muscles and perimuscular connective tissues between subjects with lumbopelvic pain and healthy subjects, did not find differences between the groups for the thickness of the TrAb, OI or EO, while they exhibited a thinner RA and a wider IRD in subjects with lumbopelvic pain. Therefore, these findings highlight the contribution of AR within the abdominal musculature and its implication in the development and persistence of lumbopelvic pain. In the Whittaker study, the inclusion criteria did not include a minimum amount of pain on the VAS scale, and a pain duration of at least six weeks was also required. Considering that PD is an intermittent chronic pain that can appear throughout a woman’s fertile life, we might think that nociceptive processing is different in both cases, opening new lines of investigation.

Primary dysmenorrhea is associated with enhanced pain sensitivity and temporal summation in adult women, which is present in PD and may reflect the presence of central pain processes [27]. This central sensitization is in relation to many factors, such as the severity of symptoms, and because of this we could conclude that our results could have been different if the inclusion criteria would have settled on a higher VAS [32].

In addition, Stuge et al. [33] examined abdominal and pelvic floor muscle function in women with and without long-term pain, finding that the thickness of the deep abdominal musculature (TrAb and IO) was similar at rest in both groups. Likewise, in another study about the morphology of abdominal muscles in ballet dancers with and without lower back pain, using MRI it was shown that the thickness of the TrAb and the resting IO did not differ between dancers with and without lower back pain [34]. Consequently, more research is needed in order to provide a clear explanation about causes and treatment of pain in PD.

### Limitations and Future Lines

The results of this study should be considered in light of the following limitations:

All data are self-reported based on a data collection form. Therefore, the information on the menstruation of the participants and their disorders could not be validated, which may be subject to information bias. In addition, the daily habits of the subjects, including smoking, alcohol consumption, medication and the amount of exercise were not controlled and may hinder the ability to explore their relationship with PD.

More research is recommended to determine the importance of considering the contribution of all muscles, as well as perimuscular connective tissue, rather than focusing solely on the components individually.

## 5. Conclusions

In conclusion, this study did not show statistically significant differences between the PD group and healthy participants in the thicknesses of the TrAb, IO, EO, RA and IRD muscles. Thus, the morphology of abdominal wall muscles was not related with PD disorders.

## Figures and Tables

**Table 1:** Demographic data of the sample.

Data	DP (*n* = 19)	Controls (*n* = 20)	*p*-Value
Age, years	20 ± 4 ^†^	22.50 ± 7 ^†^	0.647 ^††^
Height, m	1.64 ± 0.09 ^†^	1.68 ± 0.055 *	0.134 ^††^
Weight, kg	56.00 ± 23 ^†^	60.55 ± 7.29 *	0.322 ^††^
BMI (kg/m^2^)	21.61 ± 3.26 *	21.47 ± 3.67 *	0.881 *

Abbreviations: PD—primary dysmenorrhea; BMI—body mass index. * Mean ± standard deviation (SD) was applied. ^†^ Median ± interquartile range (IR) was used. ^††^ The Mann-Whitney U-test was performed.

**Table 2:** Ultrasound measurements.

Measures	PD (*n* = 19)	Controls (*n* = 20)	*p*-Value
Distance (cm) IRD	0.96 ± 0.48 (0.29–2.28) *	1.26 ± 0.59 (0.54–2.58) *	0.098 **
Thickness (cm)			
Right TrAb	0.32 ± 0.74 (0.21–0.50) *	0.34 ± 0.10 (0.18 –0.61) *	0.428 **
Right IO	0.72 ± 0.14 (0.41–0.97) *	0.71 ± 0.17 (0.41–1.09) *	0.903 **
Right OE	0.48 ± 0.09 (0.29–0.60) *	0.49 ± 0.11 (0.33–0.70) *	0.557 **
Left TrAb	0.30 ± 0.06 (0.18–0.43) *	0.32 ± 0.09 (0.20–0.57) *	0.413 **
Left IO	0.72 ± 0.14 (0.46–0.94) *	0.72 ± 0.17 (0.44–1.12) *	0.939 **
Left EO	0.53 ± 0.79 (0.40–0.69) *	0.52 ± 0.12 (0.34–0.81) *	0.930 **
Right RA	1.02 ± 0.16 (0.67–1.22) *	1.08 ± 0.21 (0.76–1.52) *	0.341 **
Left RA	1.06 ± 0.13 (0.77–1.30) *	1.07 ± 0.22 (0.78–1.54) *	0.873 **

Abbreviations: TRAB—transverse abdominal; IO—internal oblique; EO—external oblique; RA—rectus abdominis; IRD, inter-rectus distance. * Mean ± standard deviation (SD) (minimum-maximum) was applied. ** The Student *T*-test was performed for independent samples.
